# FACTORS INFLUENCING FLUOROSCOPY TIME IN ENDOVASCULAR TREATMENT OF ABDOMINAL ANEURYSMS: A RETROSPECTIVE STUDY

**DOI:** 10.1093/rpd/ncad025

**Published:** 2023-02-13

**Authors:** Fotios O Efthymiou, Stavros K Kakkos, Vasileios I Metaxas, Christos P Dimitroukas, Konstantinos G Moulakakis, Spyros I Papadoulas, Natasa K Kouri, Andreas L Tsimpoukis, Konstantinos M Nikolakopoulos, Chrysanthi P Papageorgopoulou, George S Panayiotakis

**Affiliations:** Department of Medical Physics, School of Medicine, University of Patras, Patras, Greece; Department of Vascular Surgery, School of Medicine, University of Patras, Patras, Greece; Department of Medical Physics, School of Medicine, University of Patras, Patras, Greece; Department of Medical Physics, School of Medicine, University of Patras, Patras, Greece; Department of Medical Physics, University Hospital of Patras, Patras, Greece; Department of Vascular Surgery, School of Medicine, University of Patras, Patras, Greece; Department of Vascular Surgery, School of Medicine, University of Patras, Patras, Greece; Department of Vascular Surgery, School of Medicine, University of Patras, Patras, Greece; Department of Vascular Surgery, School of Medicine, University of Patras, Patras, Greece; Department of Vascular Surgery, School of Medicine, University of Patras, Patras, Greece; Department of Vascular Surgery, School of Medicine, University of Patras, Patras, Greece; Department of Medical Physics, School of Medicine, University of Patras, Patras, Greece; Department of Medical Physics, University Hospital of Patras, Patras, Greece

## Abstract

Patients who undergo endovascular aortic aneurysm repair (EVAR) may require prolonged radiation exposure affected by several factors. The objectives of this study were to document fluoroscopy time (FT) during EVAR and identify possible factors that influence it. A retrospective analysis of a 180 patients’ database with abdominal infrarenal aortic aneurysms submitted to EVAR during a 7-y period was performed. The FT is evaluated regarding risk factors and comorbidities, graft type and patient-related, clinical and technical parameters. FT’s median (interquartile range) was 1011 (698–1500) s. Excluder and C3 Excluder were associated with significantly lower FT values when compared with other grafts. Hypertension, dyslipidemia, age ≥ 70 y, maximum aneurysm diameter ≥ 6 cm and procedure duration ≥2 h resulted in higher FT values. A significantly lower FT was found for the operations performed in the 7th y of the study’s period compared with the previous 6 y, mainly because of the use of Excluder or C3 Excluder grafts. However, these grafts did not show any significant difference in FT values during the 7 y. A significant correlation between FT with age and procedure duration was found. Nevertheless, procedure duration is a poor FT predictor in linear and logistic regressions, although is significantly correlated with FT. Dyslipidemia, procedure duration and graft type are independent predictors of FT larger than the median, whereas only the procedure duration is a predictor for FT larger than the 75th percentile value. The identified factors regarding radiation protection issues should be considered when contemplating abdominal aortic aneurysm repair, however, without compromising the procedure’s efficacy. Further work is necessary to identify more potential anatomical, clinical and technical factors affecting procedures’ complexity and FT and patient radiation dose during EVAR interventions.

## Introduction

Aneurysms in the aorta’s abdominal part constitute the most important condition that vascular surgeons are called to treat^(1)^. For clinically and anatomically suitable patients, endovascular aortic aneurysm repair (EVAR) is considered the treatment of choice^(1, 2)^.

Fluoroscopic guidance is necessary to perform EVAR procedures effectively. Navigating catheters and guidewires, evaluating vessels tortuosity, localising renal and internal iliac arteries and confirming the precise stent-graft placement require fluoroscopy. Compared with traditional open surgery repair, EVAR yields a number of benefits, including small incisions exclusively in the groin region, reduced blood loss without the need for transfusion and swifter full recovery^(3)^. Despite the advantages, fluoroscopy poses potential health hazards to patients and surgeons^(4, 5)^. Health effects of radiation, either deterministic or stochastic, combined with the increased admission rates of these procedures, underline the necessity for monitoring radiation dose to patients and medical staff inside the operating theater and raising their radiation safety awareness^(6)^.

Fluoroscopy time (FT) is a patient dose surrogate commonly used to establish guidance and action levels^(7)^. The mean FT values published in previous studies range from 8.3 to 30.4 min^(8, 9)^, whereas the mean procedure duration is about 2 h^(10, 11)^. This variation in FT reflects the necessity for optimising these procedures since prolonged radiation exposures may result in patient deterministic or stochastic effects^(12)^. Studies identifying factors that affect FT are limited. Thus, the need to investigate modifiable and uncontrolled factors that potentially relate to the increased radiation exposure in EVAR procedures is of importance^(11, 13–23)^. In addition, accumulating radiation dose during follow-up should also be considered.

This retrospective study aimed to investigate the influence of clinical, anatomical and technical parameters on FT during standard EVAR procedures.

## Materials and methods

### Study design and patient population

Clinical, anatomical and technical data were retrospectively reviewed from electronic medical records for patients with infrarenal abdominal aortic aneurysms (AAAs) who underwent standard EVAR at the Department of Vascular Surgery of the University Hospital of Patras from February 2010 to December 2016. One hundred and eighty patients (178 men, 2 women) participated in the study, having a mean age of 72.2 y (range 53–92 y). These patients were suitable candidates for elective repair of unruptured AAAs with a mean aneurysm size of 5.9 ± 1.09 cm (range 3.7–9.9 cm). Not included in the current study, there were five intraoperative conversions to open repair because of graft migration (peripheral and proximal), sac rupture and graft limb thrombosis, whereas the in-hospital mortality was 0%.

Endograft device selection was performed using patients’ computed tomography angiography (CTA) report analysis and the manufacturer’s instructions. The majority of the patients were treated either with the traditional Excluder or the C3 repositionable system (C3 GORE^®^ EXCLUDER^®^ AAA Endoprosthesis, W. L. Gore and Associates, Flagstaff, AZ, USA). The Zenith Flex^®^ (Cook Medical, Bloomington, IN, USA) was applied in patients with proximal aortic neck length ≥ 10 mm. The Treovance^®^ abdominal aortic stent-graft device (Bolton Medical, Barcelona, Spain) was used in patients with unfavorable neck angulation.

### Fluoroscopic equipment

The fluoroscopy system utilised was a mobile 12-inch image intensifier C-arm (Philips BV Pulsera, Philips Medical Systems, the Netherlands, BV). The X-ray tube was positioned underneath the operating table at a focus-to-skin distance of about 30 cm during all procedures. The automatic exposure control system controlled the tube voltage and current. In addition, image manipulation options such as image grab, subtracted fluoroscopy mode and angiographic roadmapping were used.

The majority of the exposures were in the postero-anterior projection. In addition, a small number of right anterior oblique and left anterior oblique projections were employed to localise the internal iliac arteries. Supplementary to oblique projections, the cranial tilt of the C-arm may be necessary for infrarenal aortic necks of significant angulation^(24)^. The pulsed (15 frames per second) low-dose fluoroscopy mode was mainly used. High-dose fluoroscopy mode, either pulsed (15 frames per second) or continuous (30 frames per second), was used in the case of complex clinical conditions requiring enhancement of image quality to ensure the efficacy of the procedure. Magnification was mainly performed during the retrograde contralateral gate cannulation.

The fluoroscopy system was under a periodic quality control program implemented by the Medical Physics Department of the Hospital to ensure its dosimetric and imaging performance.

### Patient and operative data

Patient information, anatomical, technical-operative and clinical data were retrieved from archived electronic medical records. These included patient demographics (age, gender), date of operation, aneurysm type, maximum aneurysm diameter, stent-graft type, contrast agent volume, FT, procedure duration, use of general anesthesia, presence of intraoperative complications, need for blood transfusion, use of proximal cuff and crossed limb (‘ballerina’) configuration, as well as risk factors and comorbidities. The risk factors included hypertension, smoking, diabetes mellitus and dyslipidemia. The comorbidities included coronary artery disease, chronic obstructive pulmonary disease, and chronic kidney and cerebrovascular diseases. The study was approved by the Ethics Committee of the University Hospital of Patras (No. 29/11-7-2018). However, written informed consent was not required because of the study’s retrospective nature.

The procedures were performed by four experienced vascular surgeons assisted by junior staff members, including a trainee. No differences in FT values were found among the vascular surgeons, thus we considered all the procedures as one sample for the analysis. The EVAR technique has been previously described by the authors^(8)^.

### Statistical analysis

Descriptive statistics (mean, median, range and interquartile range (IQR)) were used for reporting patients’ and operative data. The FT was compared with corresponding values previously published. The Kolmogorov–Smirnov goodness-of-fit test with Lilliefors significance correction was used to check the normality of the data. The Spearman Rho correlation test was used to investigate the correlation between FT and quantitative data. The Mann–Whitney and the Kruskal–Wallis tests were used to investigate the existence of a significant difference in FT values among various groups studied, as appropriate. Conover’s post hoc test was also used for pairwise comparisons between various groups to find the specific pairs that presented strong evidence of significant difference. Categorical data were analysed using the chi-squared and Fisher’s Exact tests, as appropriate. Linear regressions were performed to obtain simple linear equations to predict FT from operative data. The Pearson correlation coefficient (*r*) measures the linear correlation between the operative data and FT. The corresponding coefficient of determination (*r*^2^) measures how well each linear regression model predicts the FT. Simple logistic regressions (backward, forward, stepwise) were performed to identify independent factors related to FT exceeding the median and 75th percentile values. The Hosmer and Lemeshow test was used to assess the goodness-of-fit of the logistic regression models. Statistical analysis was performed using the SPSS v.25 software package (IBM Corp, Armonk, NY). A *p*-value < 0.05 was considered statistically significant.

## Results

In this study, patient, operative and clinical data for 180 patients who underwent fluoroscopically guided EVAR procedures were retrospectively analysed. The mean, median, range and IQR of the age, maximum aneurysm diameter, contrast agent volume, FT and procedure duration values are presented in Table 1. All the FT values were considered as a single sample since no significant differences were observed among the surgeons performed the procedures (*p* = 0.235). The wide range of FT and procedure duration values (Table 1) was mainly attributed to operative and clinical factors associated with each procedure’s complexity. Mean FT and procedure duration values are higher than the median values, indicating a right skewed distribution.

**Table 1:** Patient and operative data.

	Age (y)	Aneurysm maximum diameter (cm)	Contrast agent volume (ml)	FT (s)	Procedure duration (s)
Mean (± SD)	72.2 ± 7.57	5.9 ± 1.1	137 ± 55	1326 ± 1132	7113 ± 2477
Range	53–92	3.7–9.9	50–350	353–8400	3600–18 000
Median	72	5.5	130	1011	6600
IQR	67–78	5.2–6.4	100–170	698–1500	5400–7350

The associations between various risk factors, comorbidities, and operative and clinical data with FT are presented in Table 2. The presence of hypertension, dyslipidemia, age ≥ 70 y, maximum aneurysm diameter ≥ 6 cm and procedure duration ≥2 h resulted in 21.9–96.5% higher median FT values, whereas the procedures performed in the 7th y of the study period associated with 29.6% lower median FT values. The highest difference in FT values was found for procedure duration ≥ 2 h, since prolonged procedures are generally associated with complex clinical conditions requiring extended fluoroscopic exposures.

**Table 2:** Association between FT and patient, operative and clinical factors during EVAR procedures.

Parameters	FT (s)		
	present	absent	*p*-value
Risk factors and comorbidities			
Hypertension (*N* = 132)	1084 (755–1583)	768 (540–1231)	0.0027^a^
Smoking (*N* = 139)	1078 (673–1553)	988 (719–1304)	0.68
Diabetes mellitus (*N* = 40)	1107 (753–1490)	902 (681–1516)	0.34
Dyslipidemia (*N* = 117)	1113 (720–1567)	836 (642–1235)	0.0277^a^
Coronary artery disease (*N* = 62)	1084 (765–1680)	900 (673–1500)	0.23
Chronic kidney disease (*N* = 8)	1225 (764–2055)	995 (698–1500)	0.49
Cerebrovascular disease (*N* = 7)	1380 (721–1493)	994 (701–1507)	0.72
Chronic obstructive pulmonary disease (*N* = 21)	978 (699–1569)	1020 (694–1496)	0.96
Aorto-iliac aneurysms (*N* = 47)	988 (739–1565)	1080 (678–1489)	0.85
General anesthesia (*N* = 146)	998 (716–1527)	1111 (650–1451)	0.84
Ballerina limb configuration (*N* = 22)	1119 (758–2040)	1002 (686–1478)	0.23
Intraoperative complications^b^ (*N* = 24)	962 (608–1559)	1011 (706–1493)	0.48
Age ≥ 70 y (*N* = 113)	1080 (722–1635)	840 (606–1319)	0.0201^a^
Aneurysm maximum diameter ≥ 6 cm (*N* = 68)	1097 (802–1569)	900 (623–1478)	0.0484^a^
Blood transfusion (*N* = 10)	1534 (1020–1920)	988 (683–1474)	0.08
Contrast agent volume ≥ 100 mL (*N* = 141)	1047 (714–1580)	879 (602–1269)	0.08
Seventh year of operations (*N* = 45)	760 (597–1130)	1080 (741–1585)	0.0034^a^
Females (*N* = 2)	971 NE^c^	1011 (705–1500)	0.60
Procedure duration ≥7200 s (*N* = 84)	1493 (1144–2263)	760 (601–980)	<0.0001^a^
Proximal cuff (*N* = 12)	842 (608–1470)	1020 (706–1500)	0.52

Values are presented as median (IQR).

^a^Statistically significant.

^b^Including placement of distal extension.

^c^Not estimable.

**Figure 1:**
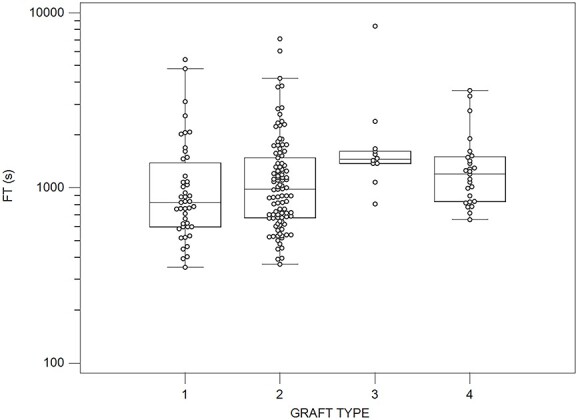
FT values for the four groups of grafts used during EVAR procedures, 1: Excluder (*N* = 43), 2: C3 Excluder (*N* = 102), 3: Zenith flex (*N* = 10) and 4: Treovance (*N* = 25) (Kruskal–Wallis test, *p* = 0.00779).

Median (IQR) FT values for four groups of graft types used are presented in Figure 1. The most frequently used graft types were the Excluder (*N* = 43) (Group 1) and C3 Excluder (Group 2) (*N* = 102), which resulted in significantly lower median FT values (median = 900 s, IQR: 645–1474 s) compared with other grafts (median = 1300 s, IQR: 924–1553 s) (*p* = 0.0024). Overall, a significant difference is found in FT for the different groups of graft types (*p* = 0.00778) used. Furthermore, post hoc analysis indicated that the pairwise comparisons that gave rise to this difference included those between Groups 1 or 2 and 3.

**Figure 2:**
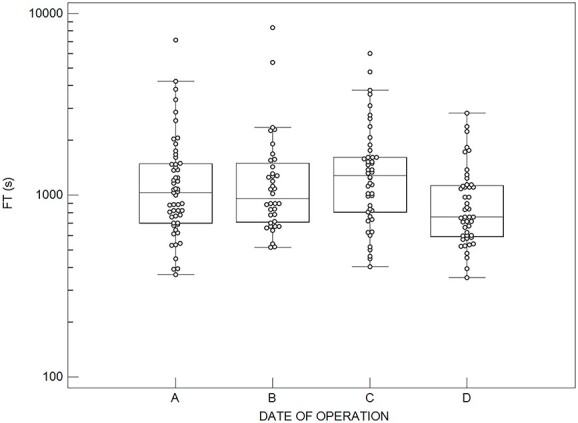
FT values for the four groups based on the date on which the EVAR operation performed, A: 2010–11 (*N* = 53), B: 2012–13 (*N* = 36), C: 2014–15 (*N* = 46) and D: 2016 (*N* = 45) (Kruskal–Wallis test, *p* = 0.0163).

A statistically significant difference was also found in median FT values among the four groups based on the operation’s date (*p* = 0.016) (Figure 2). A significantly lower FT was found in post hoc pairwise comparisons between the last year (Group D) and the previous years of operations (Groups A–C), which is attributed to the fact that during the last year, Excluder (*N* = 11) and C3 Excluder grafts (*N* = 31) were mainly used (42/45 procedures), as well as the familiarisation of the surgeons with the implementation of these grafts. Additionally, it should be noted that FT values for Excluder and C3 Excluder grafts did not show a significant difference among the Groups A–D (*p* = 0.158 and *p* = 0.176, respectively).

The correlations between FT and patient and operative data are presented in Table 3. There was a significant correlation between FT and age (rho: 0.176, *p* = 0.0179) and procedure duration (rho: 0.662, *p* < 0.0001). There was no significant correlation between FT and aneurysm maximum diameter or contrast agent volume.

**Table 3:** Correlation of FT with patient and operative data.

		Age	Aneurysm maximum diameter	Contrast agent volume	Procedure duration
FT	Correlation coefficient^d^	0.176	0.105	0.122	0.662
	*p*-value	0.0179	0.162	0.103	<0.0001

^a^Spearman test.

**Figure 3:**
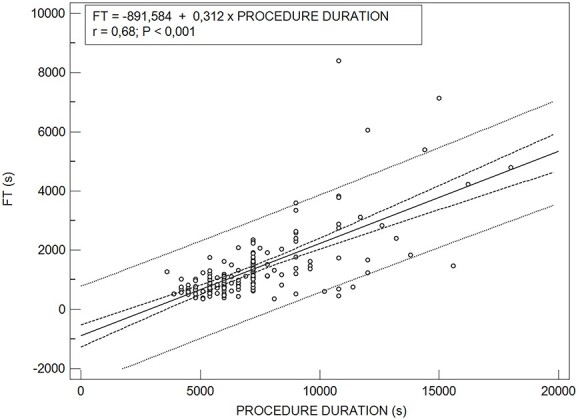
Linear regression between FT and procedure duration (*p* < 0.0001). The 95% confidence intervals are represented by the gray dotted curves and the 95% prediction interval by the black dashed curves drawn parallel to the regression line. The *r* = 0.68 is the Pearson correlation coefficient and is a measure of the correlation between the procedure duration and FT.

Figure 3 is a scatter plot of FT values including the regression line that best fits the plotted points. Simple linear regressions showed that the procedure duration is a poor predictor of FT (Figure 3). The *r* = 0.68 (*r*^2^ = 0.46) indicates that the obtained linear regression equation does not represent the data well. The 95% CI and the 95% prediction curves were also fitted. These curves represent a 95% CI and 95% prediction interval for the regression line. The CI simply provides error intervals of FT values, whereas the prediction interval reflects the error in predicting the FT for a given procedure duration value. The prediction interval showed a large variation in FT values for a single procedure duration, especially for higher procedure duration values (Figure 3). This furtherly demonstrates that the procedure duration is not sufficient as a stand-alone parameter to predict accurately the FT.

Simple logistic regressions showed that dyslipidemia, procedure duration and stent-graft type could be used as single independent predictors for FT larger than the median value. In contrast, the procedure duration is the predictor for FT larger than the 75th percentile (Table 4).

**Table 4:** Simple logistic regression models between FT and patient, operative and clinical factors during EVAR procedures.

Simple logistic regression	Logistic regression model	Regression equation significance *p*-value	Odd ratio (95% CI)	Hosmer and Lemeshow test *p*-value
Stepwise and backward for FT > median	Constant: −0.5534 Dyslipidemia:	0.0076	2.3304 (1.2408–4.3768)	1.000
	0.84605 *p* = 0.0085			
	Constant: −0.3379 Stent graft type:	0.0037	1.2046 (1.0564–1.3737)	1.000
	0.18617 *p* = 0.0055			
	Constant: −5.0059 Procedure duration:	<0.0001	1.0007 (1.0005–1.0010)	0.0005
	0.00073938 *p* < 0.0001			
Stepwise and backward for FT > 75th percentile	Constant: −5.9085 Procedure duration:	<0.0001	1.0006 (1.0004–1.0008)	0.0860
	0.00062933 *p* < 0.0001			

## Discussion

Exposure to radiation during EVARs could adversely affect medical personnel involved in the procedure and patients receiving treatment. The investigation to identify factors influencing the FT during EVARs may therefore help vascular surgeons predict which procedures are associated with prolonged FT and make appropriate precautions to avoid adverse effects because of ionising radiation^(6)^. In this study, the FT has been found to be dependent on several parameters, such as stent-graft type, risk factors and comorbidities, as well as operative and clinical factors affecting the complexity of the procedure (Table 2; Figures 1 and 2)^(14, 15, 20, 23)^. Notably, additional radiation dose is required during frequent follow-up visits, utilising CTA scans, to verify that the stent-graft continues to function properly.

Previous studies have reported patient doses and FT values during various EVAR procedures. Still, analyses of the effect of various clinical factors on FT and radiation dose have rarely been documented^(9, 11, 13, 16, 21)^. The main findings from this study suggest that FT is directly associated with several anatomical, operative and clinical factors determining each EVAR procedure’s complexity^(14, 15, 20, 23)^. More specifically, the presence of hypertension, dyslipidemia, age ≥ 70 y, aneurysm maximum diameter ≥ 6 cm and procedure duration ≥7200 s was associated with significantly increased FT (*p* < 0.05) (Table 2) since they are often related to the treatment of more demanding AAAs. Furthermore, the FT showed a strong positive correlation with procedure duration (*p* < 0.0001) (Table 3) and is a predictor of FT higher than the median and 75th percentile values in simple logistic regression models (*p* < 0.0001) (Table 4). Overall, a significant difference is found in FT values among the different graft type groups (*p* = 0.00779) (Figure 1) and date of operation groups (*p* = 0.016) (Figure 2); however, the graft type is a predictor only for FT higher than the median in the simple logistic regression model.

A large variation was observed in the median and 75th percentile FT values compared with those published in previous studies^(8)^. This variation is mainly because of the differences in patients’ characteristics, complexity of clinical conditions and the type of fluoroscopic equipment used. However, because of the lack of information in the previous studies, a direct comparison with the current study would be inappropriate.

Regarding the complexity of the EVAR procedure, Tzanis *et al*.^(14)^ proposed three complexity indices to separate straightforward procedures with favorable anatomy from the demanding ones (higher complexity score), based on several clinical factors that may result in prolonged FT values. They also reported reference (median, third quartile) FT values based on the complexity indices^(14)^. Our study’s median and third quartile FT values were 16.9 and 25 min, respectively (Table 1). Also, the presence of several different intraoperative complications, including placement of distal extensions, did not significantly affect FT (Table 2). However, a strong significant correlation of FT with procedure duration values was found (Table 3), highlighting that additional factors could contribute to the procedures’ complexity, resulting in increased procedure duration and, consequently, higher FT values because of adjunctive strategies effectively perform the operation. For procedures with a duration higher than 2 h, a 2-fold significantly higher FT is needed (Table 2). A significant correlation was also found with age (Table 3), highlighting that an older patient with advanced disease may need prolonged exposure. This is also demonstrated in Table 2, where patients with age ≥ 70 y old and aneurysm maximum diameter ≥ 6 cm showed significantly increased FT. Furthermore, this could be explained by the use of a bifurcated graft and the extra effort required for the cannulation of the contralateral limb. However, it is unclear why hypertension significantly increases the FT (Table 2), although this finding may be attributed to altered anatomy in such patients.

In a similar study, Kalef-Ezra *et al*.^(20)^ reported almost the same FT values (median, 17.2 min) (Table 1) for 91 patients who underwent AAA repair utilising a Philips Pulsera mobile C-arm system as that used in our study. Bruschi *et al*.^(23)^ reported lower FT values for EVAR procedures performed both in a conventional operating theater and an angiosuite using a low-dose protocol (‘care aorta’) (median, 11 min) or ‘standard aorta’ protocol (median, 9 min). In complex cases requiring intraoperative maneuvers, both FT and use of DSA have been increased, as well as magnification, resulting in higher patient exposures. The differences are mainly attributed to the anatomical region of the aorta where the procedure was performed, the surgical technique, and the different implementation of the fluoroscopy system. Furthermore, in this study, the surgeon controlled the C-arm system; thus, a limited number of patients received higher exposures (Figure 1)^(11, 25)^. These procedures had intraoperative complications, successfully solved with an adjunctive maneuver (proximal or distal extension). The small number of prolonged procedures is probably the main reason that the presence of intraoperative complications did not result in significantly different FT values (Table 2).

Generally, the fixed imaging systems perform well under challenging patients with higher body mass index (BMI) requiring imaging at different projection angles that might not be possible with mobile C-arm systems; however, with an increased radiation dose to the patient^(15)^. Nevertheless, Kalender *et al*.^(16)^, for an angiography suite, reported no significant correlation between FT (mean, 22.2 ± 12.3 min) and patients’ BMI. Machado *et al*.^(11)^ also reported mean FT for three groups of patients based on BMI (<25 kg/m^2^: 23.6 min, 25–30 kg/m^2^: 19.1 min, >30 kg/m^2^: 25.8 min). Advanced software for image fusion may help the surgeon during the operation and, at the same time, reduce the FT^(15)^. At this point, it should be noticed that the skills and familiarisation of the vascular surgeons with the technique allow for swifter procedures with reduced FT^(26)^. The fixed angiographic systems’ advantages have been described in detail^(23)^; however, the settings of such systems need to be improved to gain the benefits regarding dosimetric and imaging performance.

Machado *et al*.^(11)^ investigated several factors influencing the radiation exposure during EVARs performed in an operating room utilising a Philips Pulsera mobile C-arm system. They concluded that the aneurysm’s morphology, patient characteristics and the procedure’s technical difficulty were all associated with increased FT. Furthermore, they pointed out that each anatomical risk factor independently is not associated with increased FT, but only the number of factors present, regardless of which they are. Without anatomical risk factors, they reported a mean FT of 19.1 min, whereas for one or greater than two anatomical factors, a mean FT of 22.2 and 26.2 min, respectively. The mean aneurysm maximum diameter (6.1 cm) and mean FT (20.6 min) values are almost the same, whereas the mean procedure duration (103 min) was lower than in our study (Table 1). A significantly higher FT was found for the age group of <70 y (14 vs 20 min), as well as for both male (16.9 vs 22 min) and female groups (16.2 vs 19.1 min) compared with the results presented in Table 2. When evaluating the influence of an aneurysm’s diameter, a statistically significant increase in FT was found for maximum aneurysm diameter ≥ 6 cm (15 vs 18.3 min). In contrast, a significant decrease in FT (mean, 24.3 vs 19 min) was reported by Machado *et al*.^(11)^. Majewska *et al*.^(21)^ provided information about the risk factors and comorbidities of patients treated with EVAR during a 5-y period; however, no analysis was performed regarding their effect on FT and patient dose.

Concerning the different graft types used, the use of Excluder and C3 Excluder grafts are associated with lower FT values (Figure 1). A significant difference was found among the groups investigated in this study (Figure 1), which is not observed in the Machado *et al*.^(11)^ study. This finding is attributed to the easiness of deployment and the presence of one docking limb (often not requiring extension) of the Excluder or C3 Excluder grafts, or alternatively, more straightforward anatomy in combination with surgeon experience. This is further demonstrated in Figure 2, where the decrease in FT values observed during the 7th y since the most frequently used grafts during this year were the Excluder or C3 Excluder grafts. Notably, no differences were found in FT values for Excluder and C3 Excluder grafts during the entire study period. Since we have been using all graft types studied herein for several years, we cannot attribute our findings to a learning curve issue, although the maintenance of technical skills may have played a role. A problem for which it is difficult to compensate is the influence of the inevitable learning curve, as well as the evolution of stent-graft technology and technical adjuncts in fluoroscopy usage during the 7-y period.

Regarding the effect of the different grafts on FT (Figure 1), the results of this study verified the findings of a previous study conducted in our department^(9)^, which likewise demonstrated that the C3 Excluder graft resulted in significantly lower FT compared with other grafts. All the risk factors and comorbidities resulted in similar effects on FT in both studies; however, a significantly increased FT has only been reported for patients with coronary artery disease^(9)^, whereas in this study, FT was increased significantly only for patients with hypertension and dyslipidemia. These findings are probably because of the small number of patients (*N* = 11) with coronary artery disease and the increased FT values (larger IQR, 778–2748 s) mainly associated with some complex procedures requiring adjunctive maneuvers. Furthermore, the significant increase in FT values for patients with hypertension and dyslipidemia is probably because of the larger number of patients without hypertension (*N* = 48) and dyslipidemia (*N* = 63) compared with those in the previous study (*N* = 22 and *N* = 8, respectively)^(9)^, which probably did not exhibit complex anatomy and thus resulted in a relatively lower IQR (540–1231 s and 642–1235 s).

On the other hand, in some studies, the use of dedicated angiographic equipment resulted in higher FT values than our study, mainly because of patient-related factors, surgeons’ technique and skills, and imaging system-related factors^(15, 22)^. For example, Rehman *et al*.^(22)^ reported median FTs of 26.96 and 36.02 min for standard EVAR procedures (with no iliac aneurysms) performed in a dedicated vascular hybrid operating room and a conventional operating theater, respectively. They also reported a significant reduction in contrast agent volume values (114 vs 158 mL) in the hybrid compared with the conventional operating room, comparable to those in our study (Table 1). Stangenberg *et al*.^(15)^ also demonstrated a reduction of 24.55% in median FT (from 28.1 to 21.2 min) for standard EVAR procedures performed in a hybrid operating room after the implementation of advanced dose reduction technologies (ClarityIQ). In another study, van de Haak *et al*.^(27)^ reported a significant reduction in mean FT of about 50% when the ClarityIQ software was used for aortoiliac occlusive disease interventions (6.51 ± 6.80 min vs 9.82 ± 11.27 min), whereas for standard EVAR procedures the mean FT values were almost the same (13.10 ± 6.09 min vs 13.56 ± 7.85 min). However, these FT values were significantly lower than our study’s (Table 1).

Additionally, the ClarityIQ software did not significantly affect the mean procedure duration and contrast agent volume used for standard EVAR and aortoiliac interventions. However, the mean procedure duration (94.97 ± 34.19 min) and mean contrast medium volume (64.79 ± 31.09 mL) were significantly lower than those of our study (Table 1). The above may be attributed to the fewer DSA runs in cranio-caudal and oblique angulations to achieve a view of the aortic neck and iliac vessels, which is verified by the smaller volume of contrast medium used.

Ockert *et al*.^(10)^ reported that the endovascular aortic sealing (EVAS) system (Nellix Endologix Inc., Irvine, Calif) is associated with a decrease in FT and procedure duration when compared with standard EVAR. The FT is significantly correlated with patients’ weight, BMI and procedure duration, as in our study (Table 3). The mean patients’ age and aneurysm maximum diameter values are almost identical in the two studies (72.48 vs 72.2 y, 5.67 vs 5.9 cm) (Table 1). Ockert *et al*.^(10)^ also provided some information about risk factors and comorbidities in the EVAR and EVAS groups (hypertension, coronary artery disease, diabetes and renal insufficiency); however, the association of these factors with FT was not investigated as in our study (Table 2). A significant reduction of screening time and procedure duration after EVAS was also reported by Antoniou *et al*.^(28)^ utilising the same mobile C-arm system (Philips Veradius Neo). Butler *et al*.^(13)^ found that FT is significantly lower for patients who did not require post-deployment procedures (PDPs) compared with patients that needed PDPs. The contrast volume used was not significantly different between the two groups.

Linear regression analysis showed a significant association of FT with procedure duration values (Figure 3). The 95% confidence and 95% prediction intervals for the regression curve are also presented in Figure 3, along with the corresponding regression equation. The prediction interval represents the 95% probability for the FT values regarding the procedure duration values, whereas the CI includes the true regression line with 95% probability. However, it should be noted that the regression equation could only be applied for values obtained from patients with similar anatomical characteristics or under similar clinical conditions and provides a poor prediction of FT. Otherwise, FT could be underestimated or overestimated because of the differences in anatomical clinical, technical parameters affecting the complexity of each procedure.

Τhe procedures performed in the 7th y of the study period (Table 2) and the use of Excluder and C3 Excluder grafts are associated with lower FT values (Figure 1). Nevertheless, on simple logistic regression models presented in Table 4, dyslipidemia, stent-graft type and procedure duration were independent predictors of FT higher than the median value. However, the procedure duration was the only independent factor associated with FT higher than the 75th percentile. Of note, among the factors that significantly affected FT, only the procedure duration is a predictor of FT higher than median and 75th percentile values, whereas the stent-graft device is a predictor of FT higher than median only.

The main weakness of the study is the investigation of factors that affect FT instead of KAP or cumulative air kerma, which correlate better with patient and staff exposure. During the study period, FT was the only recorded dose metric regarding the patient’s exposure. Additional limitations include the study’s retrospective design and lack of data regarding BMI, and aneurysms’ morphological characteristics. In addition, this is a single-institution study where advanced imaging applications such as fusion or ClarityIQ software are not installed on the mobile C-arm used, and thus, in order to evaluate their effect on FT reduction, a prospective study in a hybrid room or an angiosuite would be needed.

## Conclusion

The main factors that significantly affect FT during standard EVAR procedures performed in an operating theater include hypertension, dyslipidemia, age ≥ 70 y old, maximum aneurysm diameter ≥ 6 cm, procedure duration ≥ 2 h, procedures performed on the 7th y of the study and use of the Excluder or C3 Excluder graft. However, these factors should be considered regarding radiation protection issues when contemplating AAA repair without compromising the procedure’s efficacy. Regression models were also developed to predict the FT, and the procedure duration was the most significant factor. Further work is necessary to identify more potential anatomical, clinical and technical factors affecting procedures’ complexity and FT and patient radiation dose during EVAR interventions.

## Data Availability

The datasets analysed during the current study are available from the corresponding author on reasonable request.
